# A Comparative Analysis of Selected Methods for Determining Young’s Modulus in Polylactic Acid Samples Manufactured with the FDM Method

**DOI:** 10.3390/ma15010149

**Published:** 2021-12-25

**Authors:** Bartosz Pszczółkowski, Konrad W. Nowak, Wojciech Rejmer, Mirosław Bramowicz, Łukasz Dzadz, Remigiusz Gałęcki

**Affiliations:** 1 Department of Material and Machine Technology, University of Warmia and Mazury in Olsztyn, ul. Oczapowskiego 11, 10-719 Olsztyn, Poland; wojciech.rejmer@uwm.edu.pl (W.R.); miroslaw.bramowicz@uwm.edu.pl (M.B.); 2 Department of Systems Engineering, Faculty of Technical Sciences, University of Warmia and Mazury in Olsztyn, ul. Jana Heweliusza 14, 10-718 Olsztyn, Poland; konrad.nowak@uwm.edu.pl (K.W.N.); lukasz.dzadz@uwm.edu.pl (Ł.D.); 3 Department of Veterinary Prevention and Feed Hygiene, Faculty of Veterinary Medicine, University of Warmia and Mazury in Olsztyn, 10-719 Olsztyn, Poland; remigiusz.galecki@uwm.edu.pl

**Keywords:** Young’s modulus, impulse excitation technique, ultrasonic testing, polylactic acid, 3D printing

## Abstract

The objective of this study was to compare three methods for determining the Young’s modulus of polylactic acid (PLA) and acrylonitrile-butadiene-styrene (ABS) samples. The samples were manufactured viathe fused filament fabrication/fused deposition modeling (FFF/FDM) 3D printing technique. Samples for analysis were obtained at processing temperatures of 180 °C to 230 °C. Measurements were performed with the use of two nondestructive techniques: the impulse excitation technique (IET) and the ultrasonic (US) method. The results were compared with values obtained in static tensile tests (STT), which ranged from 2.06 ± 0.03 to 2.15 ± 0.05 GPa. Similar changes in Young’s modulus were observed in response to the processing temperatures of the compared methods. The values generated by the US method were closer to the results of the STT, but still diverged considerably, and the error exceeded 10% in all cases. Based on the present findings, it might be concluded that the results of destructive and nondestructive tests differ by approximately 1 GPa.

## 1. Introduction

Fast and accurate determination of material parameters is a very important consideration in all fields of construction and manufacturing. The parameters of the manufactured materials should remain unchanged throughout the lifetime of engineered structures to guarantee safe installation and user safety. The introduction of modern manufacturing techniques such as 3D printing enabled the swift production of complex shapes in a short period of time. These are promising manufacturing methods for a wide range of applications, including reconstruction of unique elements of machines or the preparation of individual structures, e.g., in medicine and transplantology [1]. Many authors, by studying polymer processing technologies including 3D printing and fused deposition modeling (FDM) technology, have proven that material processing has a significant impact on the properties of the obtained models [1,2]. This is related to changes in material structure and during structure formation affected by the temperature-dependent solidification of the material. Research showed that processing temperature affects the change in the share of amorphous to crystalline structure in polylactic acid (PLA). This phenomenon may indirectly induce changes in the nano surface structure during the printing process [2]. The literature indicated that temperature also affects changes inside the polylactide structure; however, the minimum temperature to initiate this process is approximately 60 °C [3,4,5,6]. PLA is a very specific material due to its degradability. Degradation of this material typically occurs in hydrolytic environments. The first step is hydrolysis, during which oligomeric molecules and lactic acid are produced. In the second step, lactic acid is degraded to water and carbon dioxide through microbial activity. Degradation kinetics is determined by the stereochemistry of polymeric materials. Stereochemically pure poly-L-lactide (PLLA) is fully degraded over a period of several months [7]. The degradation of poly-DL-lactide (PDLLA) proceeds at a higher rate and is completed within several weeks. Stereospecific PLLA is utilized for medical and biological purposes, but it is less popular and more expensive. Racemic PDLLA is usually cheaper and more widely used in commercial applications [7,8,9]. The obtaining of individual structures is influenced by the method used to manufacture and process the material. These treatments candetermine the obtained structure of the material [7,9,10].

Based on the previously mentioned research, an attempt was made to evaluate the influence of product temperature on the mechanical properties of models produced on a 3D printer using FDM technology. Three-dimensional printing methods are currently used for rapid prototyping in object manufacturing [11]. The FDM technique is one of the most popular 3D printing methods andsupports fast production of durable models with sufficient strength. The main limitation of FDM is that only filament-type thermoplastics can be utilized in the 3D printing process [12]. One of the materials used in the FDM technology is PLA. Polylactic acid has several advantages. It is biocompatible and suitable for biomedical applications [13], and it degrades to non-toxic molecules that are eliminated from the body. An additional important aspect in relation to PLA isits possibility to degradein the natural environment [13]. It undergoes hydrolysis and biological degradation, and it is ultimately broken down into carbon dioxide and water [8]. Therefore, its use has a significant impact on reducing carbon footprints.

Young’s modulus is one of the most important parameters of structural materials. Young’s modulus can be determined with the use of static and dynamic methods. Static methods rely on measured values to determine the stress (σ)–strain (ε) characteristics of materials subjected to mechanical deformation (tension, flexion). Stress–strain curves are plotted to determine the coefficient of proportionality as a linear relationship. The value of the coefficient of proportionality is identical to the value of Young’s modulus. In the conventional approach, a static tensile test (STT) is the most accurate method for determining Young’s modulus. A static tensile test requires an adequate number of samples and testing machines, which increases analytical costs. Commonly, in case of typical construction materials, samples used in these tests are prepared by cutting from the original material, but in the case of fused deposition modeling, samples can be printed [14]. This test is also time-consuming, and some samples are irrecoverably damaged. Therefore, non-destructive testing methods involving a small number of compact analytical devices are urgently needed. Examples of such non-destructive acoustic testing methods, which are still being developed in the branch of materials science, are vibroacoustic testing (such as the impulse excitation technique, IET) and low power ultrasound (LPU) methods.

The IET is a method for determining the elastic properties and internal friction of materials. Resonation frequency is measured and used to calculate the Young’s modulus, shear modulus, Poisson’s ratio and internal friction of a material. Measurements can be conducted at a wide range of temperatures and in different environments. The sample is tapped with a small projectile, and the resulting vibrations are registered with a piezoelectric detector, a microphone and a laser vibrometer. The vibrational signal is registered in the time domain and converted to the frequency domain through Fourier transformation. Young’s modulus is calculated with Equations (1) and (2) [15,16,17,18,19,20,21].

(1) $$E=0.9465\cdot \left(\frac{mf_{f}^{2}}{b}\right)\cdot \left(\frac{L^{3}}{t^{3}}\right)\cdot T,$$

(2) $$T=1+6.585\cdot \left(1+0.0752v+0.8109v^{2}\right)\cdot {\left(\frac{t}{L}\right)}^{2}-0.868\cdot {\left(\frac{t}{L}\right)}^{4}-\left[\frac{8.340\cdot \left(1+0.2023v+2.173v^{2}\right)\cdot {\left(\frac{t}{L}\right)}^{4}}{1+6.338\cdot \left(0.1408v+1.536v^{2}\right)\cdot {\left(\frac{t}{L}\right)}^{2}}\right],$$

where:

*E*—Young’s modulus [Pa]; *m*—mass of the bar [g]; *b*—width of the bar [mm];
*L*—length of the bar [mm]; *t*—thickness of the bar [mm]; *f_f_*—fundamental flexural resonant frequency of bar [Hz]; *T*—correction factor for the fundamental flexural mode that accounts for the finite thickness of bar, Poisson’s ratio; *ν*—Poisson’s ratio.

Ultrasonic wave transition analysis can also be performed to determine elastic constants of materials including Young’s modulus. Similarly to the vibroacoustic method, ultrasonic wave transition analysis is a nondestructive testing method. The limiting factors include the working range of transducers, contact area and sample thickness. Ultrasonic flaw detectors with broadband transducers are used to determine elastic constants. Young’s modulus is calculated using Equation (3) [15,16,17,18].

(3) $$E=\frac{V_{L}^{2}\cdot \rho \left(1+\nu \right)\left(1-2\nu \right)}{1-\nu },$$

where:

*E*— Young’s modulus [Pa]; *V_L_*—ultrasonic wave speed [m/s]; *ρ*—density [kg/m^3^]
*ν*— Poisson’s ratio.

To the best of the authors’ knowledge, such sensorics methods have never been used to analyze the mechanical properties of structural materials fabricatedby additive manufacturing. To fill this knowledge gap, the aim of this study was to examine and develop a rapid and non-destructive method for analyzing and verifying the mechanical properties of material created viaadditive manufacturing (in particular fused deposition modeling—FDM) with the use of mobile measuring devices [11,12,22].

## 2. Materials and Methods

### 2.1. Sample Preparation

**Figure 1:**
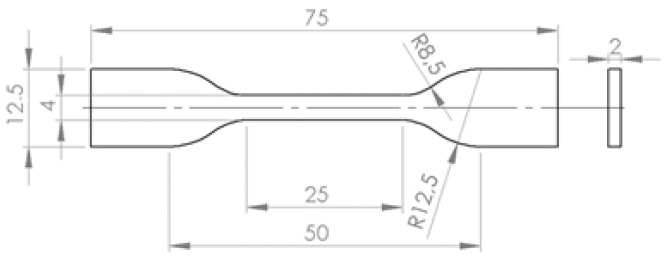
Outline of samples utilized in the static tensile material test.

The samples were manufactured from commercially available PLA filament with a diameter of 1.75 mm using the FDM technique. The following printing parameters were applied: nozzle diameter—0.4 mm, platform temperature—50 °C, single layer thickness—0.2 mm, printing speed—1800 mm/min. Extruder temperature was the variable parameter. Based on a review of the literature and the manufacturer’s recommendations, the temperature range was set as 180–230 °C. Groups of samples were manufactured at temperature increments of 10 °C (180 °C, 190 °C, 200 °C, 210 °C, 220 °C, 230 °C) based on a review of the literature [11,23,24]. Three different sample types were prepared, the sample size for every material was consistent, and every test was performed six times. Types of samples used for tensile tests are presented in Figure 1, and dimensions of rectangular samples used for density, ultrasonic and IET testing are described in following sections. 

Samples ofacrylonitrile butadiene styrene (ABS) were produced at a temperature of 240 °C with a table temperature of 80 °C. These parameters were selected on the basis of the filament manufacturer’s recommendations; the rest of the parameters remained the sameas in the case of PLA. The tests were performed in order to validate the tests performed on PLA. The processing temperature did not significantly affect the structural changes to the material, which was verified by differential scanning calorimetry (DSC) analysis and compared with the literature [25,26].

### 2.2. Ultrasonic Tests

Rectangular samples measuring 80 × 35 × 9 (±0.25) mm were prepared for ultrasonic (US) tests. Ultrasonic tests were conducted with the use of anOlympus EPOCH 4 (Olympus, Tokyo, Japan) apparatus equipped with 4 MHz dual element transducers. Ultrasonic wave propagation time was measured along the thickness of the sample (9 mm) in six replications. To facilitate the analysis, it was assumed that the studied material was rarely homogeneous.

### 2.3. IET Tests

Rectangular samples measuring 80 × 35 × 9 (±0.25) mm were prepared for tests involving the IET. Measurements were performed with anIMCA RFDA professional apparatus (IMCA, London, Assenede, Belgium). Sample mass was determined using the AS 60/220 R2 analytical scale (Radwag, Radom, Poland).

### 2.4. Tensile Tests

Sampleswith normalized shape and dimensions [27,28,29] were used in STT (Figure 1). The tests were performed with aTA.HD.plus texture analyzer (Stable Microsystems, Surrey, UK). Five samples were prepared for every nozzle temperature. None of the samples had visible fractures or other significant manufacturing flaws. The samples were subjected to tensile stress and were stretched along the long axis with constant deformation speed of 5 mm·s^−1^ until fracture occurred. 

Young’s modulus was calculated as stress Δ*σ* divided by strain Δ*ε* as a specific form of Hooke’s law, where the stress–strain curve is a linear function. Young’s modulus is the tangent to the slope of the stress–stain curve in the linear range (4).

(4) $$E=\frac{\Delta \sigma }{\Delta \varepsilon_{sp}}=tg\alpha ,$$

**Figure 2:**
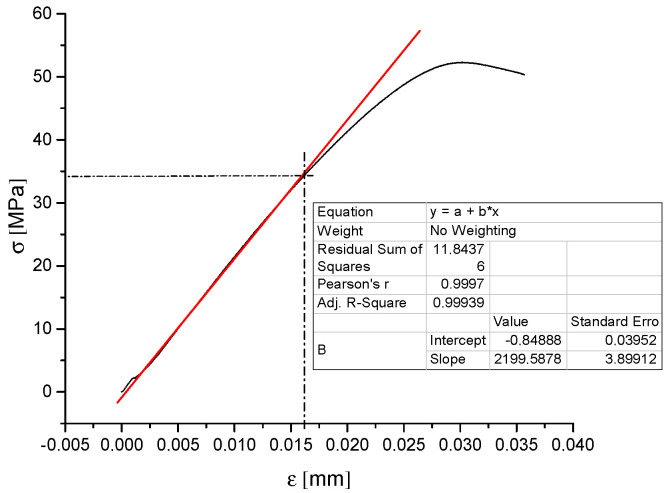
Exemplary result of stress–strain curve analysis in Origin software.

In this study, Young’s modulus was calculated by analyzing stress–strain curves with OriginPro software (OriginLab Corporation, Northampton, MA, USA). An exemplary result of the analysis is presented in Figure 2. Identical samples were used for IET and ultrasonic measurements.

### 2.5. Density Determination

Real density was determined witha HumiPyc™ Model 1 gas pycnometer (InstruQuest Inc. Scientific Instruments R & D, Boca Raton, FL, USA). The carrier gas was helium atroom temperature (22 ± 0.1 °C), and cell pressure was 220 kPa. The test involved cubical samples measuring 20 × 20 × 20 mm. Pre-test stabilization time was 10 min. Each sample was measured in five replicates.

### 2.6. Statistical Analysis

The results were processed statistically in Statistica v. 13.3 (StatSoft Inc., Palo Alto, CA, USA). Data distribution was checked with the Shapiro–Wilk test. The significance of differences between the results of the compared methods was assessed with the Kruskal–Wallis non-parametric ANOVA. Significant differences between obtained results from three methods were calculated using Tukey’s test. Differences where the *p*-value was less than 0.05 were considered significant.

## 3. Results and Discussion

The values of Young’s modulus calculated with the use of the compared methods (ultrasonic method, US; impulse excitation technique, IET; static tensile test, STT) are presented in Table 1.

The density results were relatively consistent with typical density values provided by the PLA producer (1.24 g·cm^−3^); however, ABS density (1.03 g·cm^−3^) was lower than the reference value of 1.11 g·cm^−3^ [11,30]. It can be noted that the higher the nozzle temperature, the lower the density of the printed sample. Thismaybe caused by thermal expansion of overlapping printed fibers, which then solidify faster than they contract, and couldresult in lessmass packed into the same volume.

**Figure 3:**
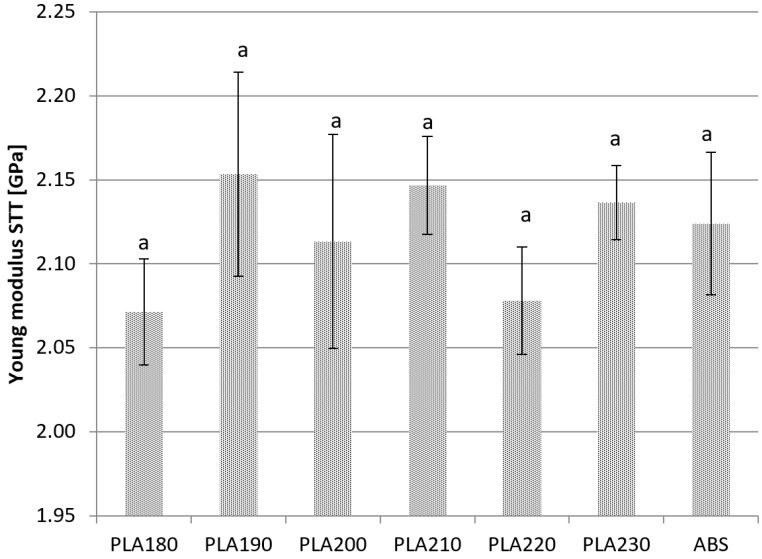
Young’s modulus values measured with STT. Bars with the different letters (a) are significantly different.

Using the STT method (Figure 3), the values of Young’s modulus were highest at 2.15 ± 0.05 GPa and 2.15 ± 0.03 GPa for PLA samples manufactured at nozzle temperatures of 190 °C and 210 °C, respectively. The lowest value of Young’s modulus was determined to be 2.06 ± 0.03 GPa on average in samples manufactured at 220 °C. In these samples, the lowest value of Young’s modulus was 2.04 GPa and the highest value was 2.16 GPa. The values calculated for the samples manufactured at 190 °C and 200 °C were characterized by the highest standard deviation. Significant differences were not observed between Young’s modulus values measured with STT.

There is a general scarcity of published studies on the 3D printing of PLA filaments, and the values of Young’s modulus for objects manufactured at 180 °C were presented in only one article [31]. The published data were significantly below (0.969 ± 0.164 GPa) the values calculated in the present study, which could be attributed to the fact that much coarser samples were used in the cited work. Another example of mechanical testing of PLA samples manufactured atsome of the temperatures used in this work was carried out by Belarbi et al. [11], where, among others, one can find strength charts of PLA samples manufactured at 200 and 210 °C. However, the authors didnot provide Young’s modulus values to compare the results. The results of the STT were used as reference values for the remaining tests, which is part of a standard procedure for determining Young’s modulus.

**Figure 4:**
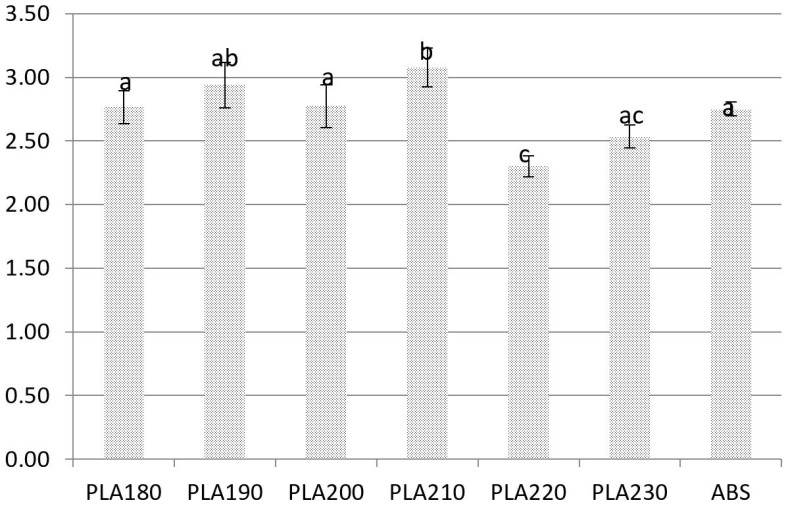
Young’s modulus values measured by US. Bars with the different letters (a, b, c) are significantly different.

The US test yielded higher results than the STT for both PLA and ABS. The lowest values of Young’s modulus were determined to be 2.30 ± 0.13 GPa in the samples manufactured at a nozzle temperature of 220 °C. The highest values were determined to be 3.08 ± 0.18 GPa in the samples printed at a nozzle temperature of 210 °C. Standard deviations decreased in samples processed at temperatures higher than 200 °C, which could be attributed to an increase in material homogeneity (Figure 4). An increase in material homogeneity could result from changes in the ratio of amorphous and crystalline phases, which was discussed by Coppola in a study of the mechanical parameters of 3D printing [23,32]. According to Lee and Liu, the cooling rate in a forced ventilation system can affect the strength and quality of prints [33].

Young’s modulus values measured with the US method can be divided to three groups based on significant differences. The first group consisted of ABS and PLA samples manufactured at nozzle temperatures of 180 °C and 200 °C. The second group included PLA samples printed at 210 °C. The third group consisted of samples obtained during additive manufacturing at 220 °C. Samples obtained with nozzle temperaturesat 190 °C and 230 °C exhibited values between the first and second and first and third groups respectively.

**Figure 5:**
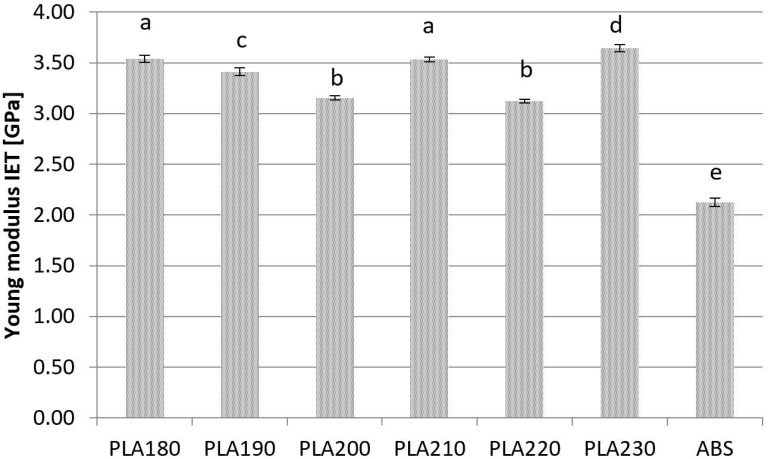
Young’s modulus values measured by IET. Bars with the different letters (a, b, c, d, e) are significantly different.

The highest values of Young’s modulus of PLAwere generated in the IET test. The lowest values were determined at 3.12 ± 0.02 GPa in the samples printed at a nozzle temperature of 220 °C. The highest values were determined at 3.64 ± 0.03 GPa in the samples manufactured at a nozzle temperature of 230 °C. The Young’s modulus measured for ABS using IET was the lowest at a value of 2.03 ± 0.03 (Figure 5).

In general, the IET test produced the highest values of Young’s modulus for PLA and the lowest for ABS. For PLA the lowest values were the reference values from the STT (Figure 5). Standard deviations were highest in the US test, which could be attributed to the fact that ultrasonic measurements are sensitive to structural anisotropy. The differences between the compared methods were statistically significant at each processing temperature (*p* < 0.05). In US tests, standard deviation was calculated based on two measurements: ultrasonic wave speed and density. The results noted in both tests were added, which could explain the high standard deviation values in the US tests. For measured values of Young’s modulus with the IET method the largestnumber of significantly different groups was observed. PLA samples obtained during printing with nozzle temperatures at 190 °C and 230 °C, and ABS samples, were significantly different from other samples. PLA samples printed at 180 °C and 210 °C did not exhibit significant differences from each other, but were different from 200 °C and 220 °C PLA samples. 

**Figure 6:**
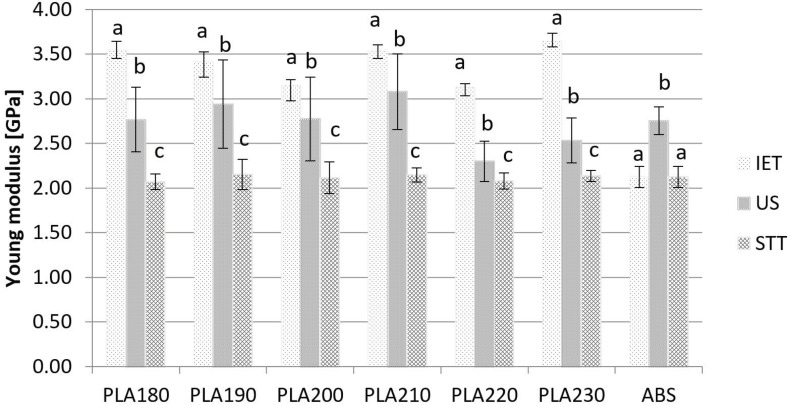
A comparison of statistical differences between Young’s modulus values calculated usingthe static tensile test and the analyzed non-destructive methods of determining Young’s modulus (STT, US and IET). Bars with the different letters (a, b, c) are significantly different.

The differences in the results produced by the compared investigated methods are shown in Figure 6. Significant differences were calculated between results obtained by three methods for a given material type. For PLA samples the results of all methods were significantly different from oneanother. Measurements of ABS gave significantly different results only for the Young’s modulus measured with the US method. The results of the US test were most similar to the reference values from STT. The greatest similarities were observed in samples produced at higher nozzle temperatures. Relative error was lowest (11.7%) in the US test of samples printed at 220 °C. The highest errors were noted in samples manufactured at 180 °C and 230 °C (71.0% and 70.0%, respectively). With the IET method, a correction factor had to be applied to the studied materials. Without the correction factor, the US method would generate more accurate results than the IET approach. Nevertheless, even the US method generated unsatisfactory results with error values in excess of 10% in all cases.

It is worth mentioning that the methods which relied on acoustic wave propagation (US and IET) produced higher values for elastic coefficients than STT, which could be explained by the fact that both US and IET are dynamic methods. The above applies to porous heterogeneous materials, including the PLA samples obtained by FDM 3D printing [18,34,35,36]. The observed phenomenon may be attributed to sample relaxation during static tensile testing. In tests that rely on vibroacoustic methods, the duration of the forces that produce a response in the analyzed material is shorter than the relaxation time. Therefore, Young’s modulus could be more accurately measured in samples that do not undergo relaxation [36]. In the IET test, the duration of the applied force was even shorter than in the US test, and the measured values of Young’s modulus were even higher. Changes in the ratio of morphological phases exert different effects on the results of US and IET tests because amorphous and crystalline phases are characterized by different vibroacoustic wave permeability. The relationship between acoustic wave permeability and temperature was explored by Mukherjee et al. [37] in a study investigating the ultrasonic properties of glass-based amorphous materials. Structural alignment was found to be directly correlated with temperature. Observation of the presented results for PLA materials can lead to the assumption that both acoustic methods (IET and US) were more susceptible to morphological changes in the material. Results for mechanical testing were not significantly different because a slow deformation rate can allow for mobility in the polymeric chains and changes of morphology during testing. The IET and US methods do not change materials’ structure, hence the significant differences in the results. It can be assumed that recrystallization occurred and the phase ratio changed when the processing temperature exceeded 200 °C. This effect was described in the calorimetric research conducted by Coppola et al. [23]. The present results show that samples obtained from printing with nozzle temperatures of 200 °C and 210 °C in both tests showed significant differences in comparison to other materials. In a study of polyethylene samples, Adachi et al. demonstrated that temperature influences ultrasonic wave transmission velocity and non-destructive measurements. The cited authors reported a correlation between polymer chain structure and crystallite morphology thatinfluences acoustic propagation [38,39]. The described phenomena were confirmed by Peng et al. during acoustic wave measurements [40]. Peng et al. found that changes in the ratio of amorphous and crystalline phases induced changes in ultrasonic wave velocity. Measurements of amorphous polymers can be less accurate than measurements of crystalline polymers because sound waves are scattered by grain boundaries. Highly crystalline polymers can be regarded as molecular composite materials where the amorphous phase is the matrix and the crystal phase is the filler [40,41,42]. In the case of the present results, fully amorphous ABS exhibited identical results inSTT and IET tests, but the results of US testing were significantly different. In the case of more crystalline PLA, IET results were the most different from the STT method. Thisresults from polyesters’ mixed morphology, where STT leads to structural changes during testing. Therefore, IET testing was more strongly influenced by the amorphous phase due to the impulsive characteristic of the test.

## 4. Conclusions

The present study demonstrated that various testing methods produce different values of Young’s modulus in PLA FDM 3D-printed samples, and partially in ABS (IET). It is assumed that the differenceswere the result of the different morphological structures of tested materials. For amorphous–crystalline materials there was a clear difference between all three methods, whilein case of a uniformly amorphous material (ABS) only the US method provided significantly different results. The results of this study indicate that the accuracy of non-destructive methods can be improved by introducing a correction factor that accounts for the ratio of morphological phases. The present findings contribute valuable information for the further development of non-destructive US tests for polymers.

## Figures and Tables

**Table 1:** Measured density and values of Young’s modulus calculated with the use of the compared methods for samples printed at all nozzle temperatures, with standard deviation values.

Nozzle Temperature [°C]	Density [g·cm^−3^]	Young’s Modulus in STT Method [GPa]	Young’s Modulus in US Method [GPa]	Young’s Modulus in IET Method [GPa]
180	1.269 ± 0.004	2.07 ± 0.03	2.76 ± 0.21	3.54 ± 0.04
190	1.258 ± 0.012	2.15 ± 0.05	2.94 ± 0.29	3.41 ± 0.04
200	1.254 ± 0.012	2.12 ± 0.07	2.77 ± 0.27	3.15 ± 0.02
210	1.254 ± 0.001	2.15 ± 0.03	3.08 ± 0.18	3.53 ± 0.02
220	1.251 ± 0.001	2.06 ± 0.03	2.30 ± 0.13	3.12 ± 0.02
230	1.246 ± 0.003	2.14 ± 0.02	2.53 ± 0.14	3.64 ± 0.03
ABS	1.026 ± 0.018	2.12 ± 0.03	2.75 ± 0.06	2.03 ± 0.03

## Data Availability

The original contributions presented in the study are included in the article. Further inquiries can be directed to the corresponding author.
